# Transfection of T-Box Transcription Factor *BRACHYURY* and *SOX2* Synergistically Promote Self-Renewal and Invasive Phenotype in Oral Cancer Cells

**DOI:** 10.3390/ijms19113620

**Published:** 2018-11-16

**Authors:** Naonari Akimoto, Kodai Nakamura, Hiroshi Hijioka, Kenichi Kume, Yoshiaki Matsumura, Tsuyoshi Sugiura

**Affiliations:** 1 Section of Oral and Maxillofacial Surgery, Division of Maxillofacial Diagnostic and Surgical Sciences, Faculty of Dental Science, Kyushu University, Fukuoka 812-8582, Japan; naonari.ocean@gmail.com; 2 Department of Maxillofacial Diagnostic and Surgical Science, Field of Oral and Maxillofacial Rehabilitation, Graduate school of Medical and Dental Sciences, Kagoshima University, Kagoshima 890-8544, Japan; pero-pulse-4.14@true.ocn.ne.jp (K.N.); zio@dent.kagoshima-u.ac.jp (H.H.); kkume@dent.kagoshima-u.ac.jp (K.K.); m.yoshiaki0424@gmail.com (Y.M.)

**Keywords:** *BRACHYURY*, *SOX2*, Epithelial–Mesenchymal transition (EMT), cancer stem cell, oral cancer

## Abstract

Recent studies suggest that epithelial–mesenchymal transition (EMT) correlates with cancer metastasis. In addition, there is growing evidence of the association of EMT with cancer stem cells (CSCs). Recently, we showed that the T-box transcription factor *BRACHYURY* could be a strong regulator of EMT and the CSC phenotype, which were effectively suppressed by a *BRACHYURY* knockdown in an adenoid cystic carcinoma cell line. In this study, we further tested whether *BRACHYURY* is a regulator of cancer stemness by means of forced expression of *BRACHYURY* in oral cancer cell lines. *BRACHYURY*, *SOX2*, or both were stably transfected into oral carcinoma cell lines. We analysed these transfectants with respect to self-renewal phenotypes using a sphere-formation assay, and we assessed the expression levels of EMT markers and stem cell markers using real-time reverse transcription-polymerase chain reaction (RT-PCR). Cell migration and invasiveness in vitro were evaluated using a wound healing assay and a tumour cell dissemination assay, respectively. Forced expression of *BRACHYURY* or *SOX2* slightly increased expression of EMT and stem cell markers and the self-renewal phenotype. The expression levels, however, were much lower compared to those of cancer stem cell-like cells. Forced co-expression of *BRACHYURY* and *SOX2* strongly upregulated EMT and stem cell markers and the self-renewal phenotype. Cell migration and invasiveness in vitro were also remarkably enhanced. These synergistic effects increased expression levels of *FIBRONECTIN*, *SNAIL, SLUG*, *ZEB1*, and *TGF-β2*. In particular, the effects on *FIBRONECTIN* and *TGF-β2* were significant. We found that *BRACHYURY* and *SOX2* synergistically promote cancer stemness in oral cancer cells. This finding points to the importance of gene or protein networks associated with *BRACHYURY* and *SOX2* in the development and maintenance of the CSC phenotype.

## 1. Introduction

Metastasis is a multistep cascade involving the migration of tumour cells from their site of origin, evasion from host defence systems, subsequent seeding at distant organs, and growth of secondary tumours. Recent studies have revealed that a specific change in the cancer cell phenotype, epithelial–mesenchymal transition (EMT), is involved in the metastasis mechanism. Accumulating evidence supports the importance of EMT in cancer progression and metastasis in several types of cancer: head and neck, breast, lung, and prostate cancer [1,2,3,4]. We also previously reported clinical evidence that EMT strongly correlates with poor prognosis in patients with oral squamous cell carcinoma [5]. In order to spawn macroscopic metastases, which are often facilitated by EMT, disseminated cancer cells presumably need a self-renewal ability similar to that exhibited by stem cells. From this point of view, the EMT process, which allows for migration and metastasis of cancer cells, would be also the key of a self-renewal capability to disseminated cancer cells. Actually, the accumulated evidences of relationship between EMT and cancer stem cells (CSCs) was reported recently [3,6,7,8,9,10,11]. The EMT or CSC program appears to be controlled by genes normally expressed in early embryos, including *TWIST, SNAIL, SLUG, ZEB1, and SOX2* [12,13,14,15]. These genes encode the transcription factors which enables the tumour cells to migrate and invade like mesenchymal cells. CSCs were proved to be resistant to chemotherapy and radiotherapy [16,17], and CSCs should be the real target for the future new concept of cancer therapies [18,19].

The T-box transcription factor *BRACHYURY* has been reported as a key gene for mesoderm formation during embryonic stage [20]. Recently, *BRACHYURY* is also recognised to induce EMT in human carcinoma cell lines [21]. In our previous study, we demonstrated that a CSC-like cell line (adenoid cystic carcinoma), ACCS-M GFP undergoes EMT [22], and that small hairpin RNA (shRNA) silencing of *BRACHYURY* downregulates EMT (and stem cell markers) in that cell line and leads to a loss of CSC-like and EMT characteristics of this cell line [11]. Tumourigenesis and metastasis of ACCS-M GFP in vivo were inhibited completely by *BRACHYURY* knockdown and partially by knockdown of *SOX2*, the conventional CSC regulator gene [11]. These data suggest that EMT may be directly linked to CSCs, and that *BRACHYURY* controls EMT and the CSC phenotype (cancer stemness). We also confirmed that expression of Brachyury protein strongly correlates with EMT and poor prognosis in oral cancer patients [5].

In this regard, *BRACHYURY* silencing could effectively control cancer stemness and offer a new concept for the development of cancer treatments. In this study, we used forced expression of *BRACHYURY* and *SOX2* in oral cancer cell lines to confirm that *BRACHYURY* and *SOX2* are regulators of the CSC phenotype.

## 2. Results

### 2.1. Forced Expression of BRACHYURY Does Not Promote Self-Renewal Capacity, But a BRACHYURYy Knockdown Suppresses the Self-Renewal Capacity in Oral Cancer Cell Lines

**Figure 1:**
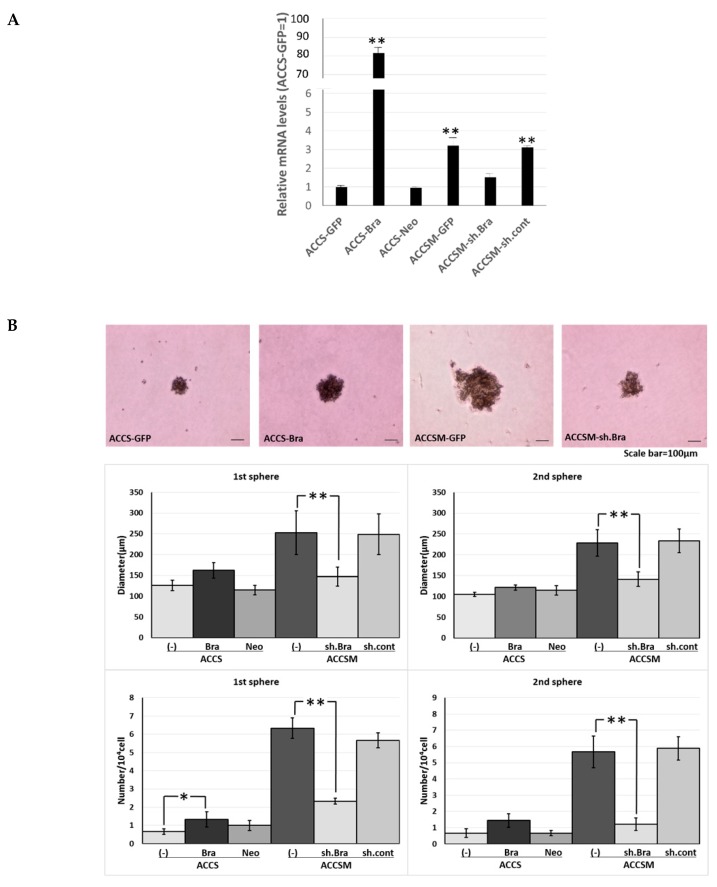
Effects of *BRACHYURY* transfection on the sphere-forming ability of ACCS (adenoid cystic carcinoma) cells. Brachyury mRNA expression levels of the indicated ACCS (adenoid cystic carcinoma) cells and in derivative clones [ACCS-Brachyury (Bra), ACCS-Neomycin (Neo), ACCSM-sh.Brachyury (sh.Bra), and ACCSM-sh.control (sh.cont)] were quantified using real-time RT-PCR. mRNA level was compared with that in ACCS-GFP cells (parental cell line), and the data are shown in arbitrary units as relative mRNA levels (ACCS-GFP = 1.0) (**A**). ACCS cells were cultured at a density of 5 × 10^4^ cells/mL in a serum-free medium for floating culture for 10 days (primary spheres). Primary spheres (day 10) were dissociated into individual cells and further cultured at a density of 10^4^ cells/mL for 10 days. The spheres were observed under a phase contrast microscope ((**B**), top panel). Sphere diameters were measured ((**B**), middle panel), and numbers (diameter > 100 μm) ((**B**), bottom panel) were counted. Sphere numbers were standardised as a sphere number per 10^4^ cells originally seeded ((**B**), bottom panel). The experiments were performed in triplicate, and the data were calculated as mean ± SD. Statistical significance of differences was analysed using the Student’s *t* test. * *P* < 0.05, ** *P* < 0.01*.*

We previously reported successful isolation of highly metastatic and tumourigenic CSC-like cells—the ACCS-M GFP cell line—from non-metastatic (0% incidence) and low tumourigenic (22.2% incidence) parental adenoid cystic carcinoma ACCS GFP cells using in vivo selection [22]. We also showed that *BRACHYURY* knockdown completely inhibits CSC and EMT phenotypes of the ACCS-M GFP cells. These findings support a crucial role of *BRACHYURY* in the regulation of cancer stemness in adenoid cystic carcinoma cell lines [11]. Therefore, in the present work, we tested the hypothesis that Brachyury can promote CSC features in adenoid cystic carcinoma cells. For this purpose, we established stable Brachyury transfectants of ACCS-GFP and ACCS-Bra cell lines. We also confirmed the effect of a *BRACHYURY* knockdown on ACCS-M GFP cells by means of Brachyury shRNA (Figure 1A). Forced expression of *BRACHYURY* slightly increased (2.0-fold) sphere formation (the number of spheres) in the primary sphere assay in comparison to parental ACCS-GFP cells (*P* = 0.0983, ANOVA), but had no effect in the secondary sphere assay (*P* = 0.125, ANOVA). In contrast, the *BRACHYURY* knockdown on ACCS-M GFP cells remarkably inhibited sphere formation in both the primary (*P* = 0.0001, ANOVA) and the secondary assay (*P* = 0.0001, ANOVA), with respect to both the diameter and the number of spheres (Figure 1).

**Figure 2:**
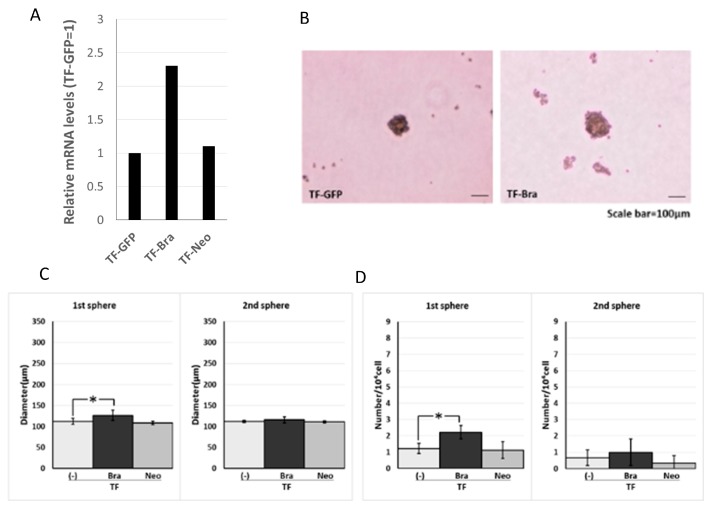
Effects of *BRACHYURY* transfection on the sphere-forming ability of TF (squamous cell carcinoma) cells. Brachyury mRNA expression levels of the indicated TF-GFP cells and in derivative clones [TF-Brachyury (Bra), TF-Neomycin (Neo)] were quantified using real-time RT-PCR. mRNA level was compared with that in TF-GFP cells (parental cell line), and the data are shown in arbitrary units as relative mRNA levels (ACCS-GFP = 1.0) (**A**). Sphere-forming ability of TF-Brachyury (Bra) and TF-Neomycin (Neo) cells were analysed and quantified as described in Figure 1. Image of phase contrast microscope (**B**). Sphere diameters (**C**), Sphere numbers (**D**). The experiments were performed in triplicate, and the data were calculated as mean ± SD. Statistical significance of differences was analysed using the Student’s *t* test. * *P* < 0.05, ** *P* < 0.01.

We next analysed the effect of Brachyury on the sphere-forming ability using another type of oral carcinoma cell, squamous cell carcinoma (SCC). For this purpose, we used SCCTF cell lines with Green Fluorescence Protein (GFP) expression (TF-GFP), *BRACHYURY* stable transfectant TF-GFP cells (TF-Bra), and control vector-transfected and selected by Neomycin (TF-Neo). Forced expression of Brachyury slightly induced (1.8-fold) sphere formation (the number of spheres) in the primary sphere assay compared to the parental TF-GFP cells, but had no effect in the secondary sphere assay (Figure 2). This finding is in agreement with the results obtained using the adenoid cystic carcinoma cells described above. We confirmed the cell proliferation rate on cell culture dish did not change in both cell lines (data not shown).

### 2.2. Forced Expression of BRACHYURY Weakly Induces EMT-Related Markers and Stem Cell Markers in Oral Cancer Cell Lines

**Figure 3:**
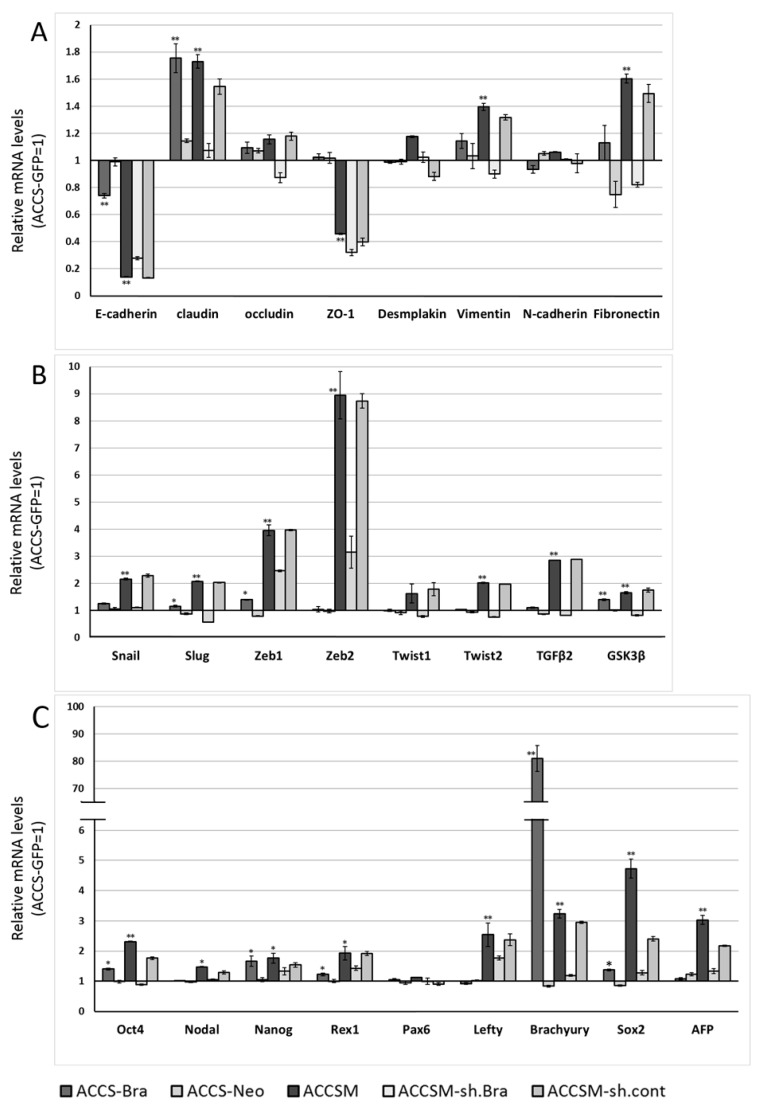
Effects of *BRACHYURY* transfection on gene expression related to epithelial–mesenchymal transition (EMT) and the cancer stem cell (CSC) phenotype in ACCS cells. mRNA expression levels of the indicated genes in ACCS cells and in derivative clones were quantified using real-time RT-PCR as described in method section. Each mRNA level was compared with that in ACCS-GFP cells (parental cell line), and the data are shown in arbitrary units as relative mRNA levels (ACCS-GFP = 1.0). The expression levels of EMT-related genes (**A**,**B**), stem cell markers, and differentiation markers (**C**) are shown. The experiments were performed in triplicate, and the data were calculated as mean ± SD. Statistical significance of differences was analysed using the Student’s *t* test. * *P* < 0.05, ** *P* < 0.01.

**Figure 4:**
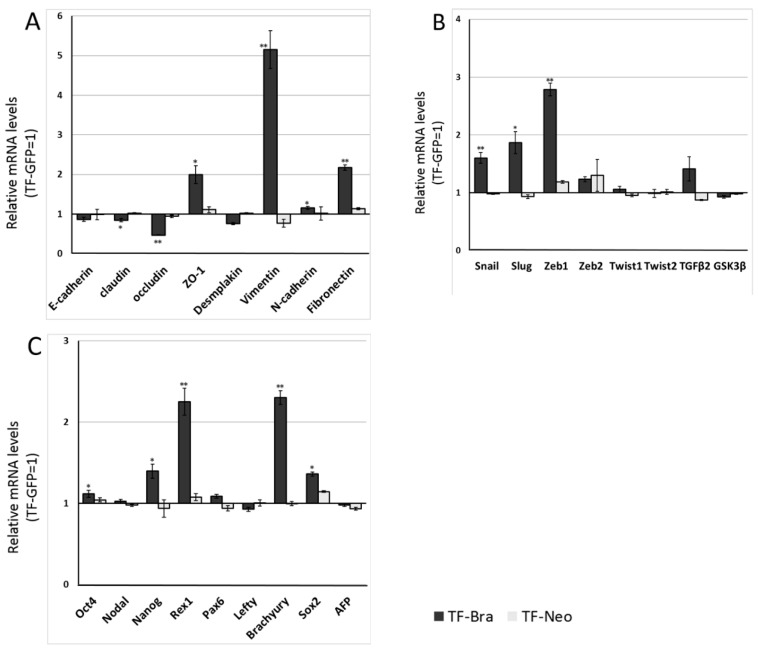
Effects of *BRACHYURY* transfection on gene expression related to epithelial–mesenchymal transition (EMT) and cancer stemness in TF cells. The mRNA expression levels of the indicated genes in TF cells and derivative clones were quantified by means of real-time RT-PCR. Each mRNA level was compared with that of TF-GFP cells, and the data are shown in arbitrary units as relative mRNA levels (TF-GFP = 1.0). The expression levels of EMT-related genes (**A**,**B**), stem cell markers, and differentiation markers (**C**) are shown. The experiments were performed in triplicate, and the data were calculated as mean ± SD. Statistical significance of differences was analysed using the Student’s *t* test. * *P* < 0.05, ** *P* < 0.01.

We next analysed the effect of *BRACHYURY* expression on EMT-related markers and stem cell markers in ACCS (Figure 3) and TF (Figure 4) cell lines. EMT and CSC markers were evaluated their expression level using quantitative real-time RT-PCR (Figure 3). Each mRNA level was compared with that in the parental cells, ACCS GFP and TF-GFP cells, and the data are shown as relative mRNA levels (ACCS GFP or TF-GFP levels are set to 1.0 arbitrary unit). We analysed the expression levels of EMT-related genes (Figure 3A and Figure 4A), stem cell markers (Figure 3B and Figure 4B), and differentiation markers (Figure 3C and Figure 4C). In ACCS cells, forced expression of *BRACHYURY* increased expression of *CLAUDIN*, *OCCULUDIN*, *VIMENTIN*, *FIBRONECTIN*, *SLUG*, *ZEB1*, *GSK3β*, *OCT4*, and *NANOG* and caused a decrease in *E-CADHERIN* expression. These changes were similar to the changes observed in the CSC-like cell line ACCS-M GFP in all markers, but the changes were smaller in *BRACHYURY* transfectants (except for *CLAUDIN*, *OCCLUDIN*, and *NANOG*). In contrast, mRNA levels of all markers decreased significantly in the *BRACHYURY* knockdown cells (ACCS-M shBra) compared to parental ACCS-M GFP cells. In TF (squamous cell carcinoma) cells, forced expression of *BRACHYURY* showed nearly the same changes in gene expression as in ACCS-M GFP cells. Notably, the increase of vimentin expression as a result of *BRACHYURY* transfection was significantly higher compared to the other markers that increased in TF-Bra cells.

### 2.3. Forced Co-Expression of BRACHYURY and SOX2 Induced the Self-Renewal Phenotype in Oral Cancer Cell Lines

**Figure 5:**
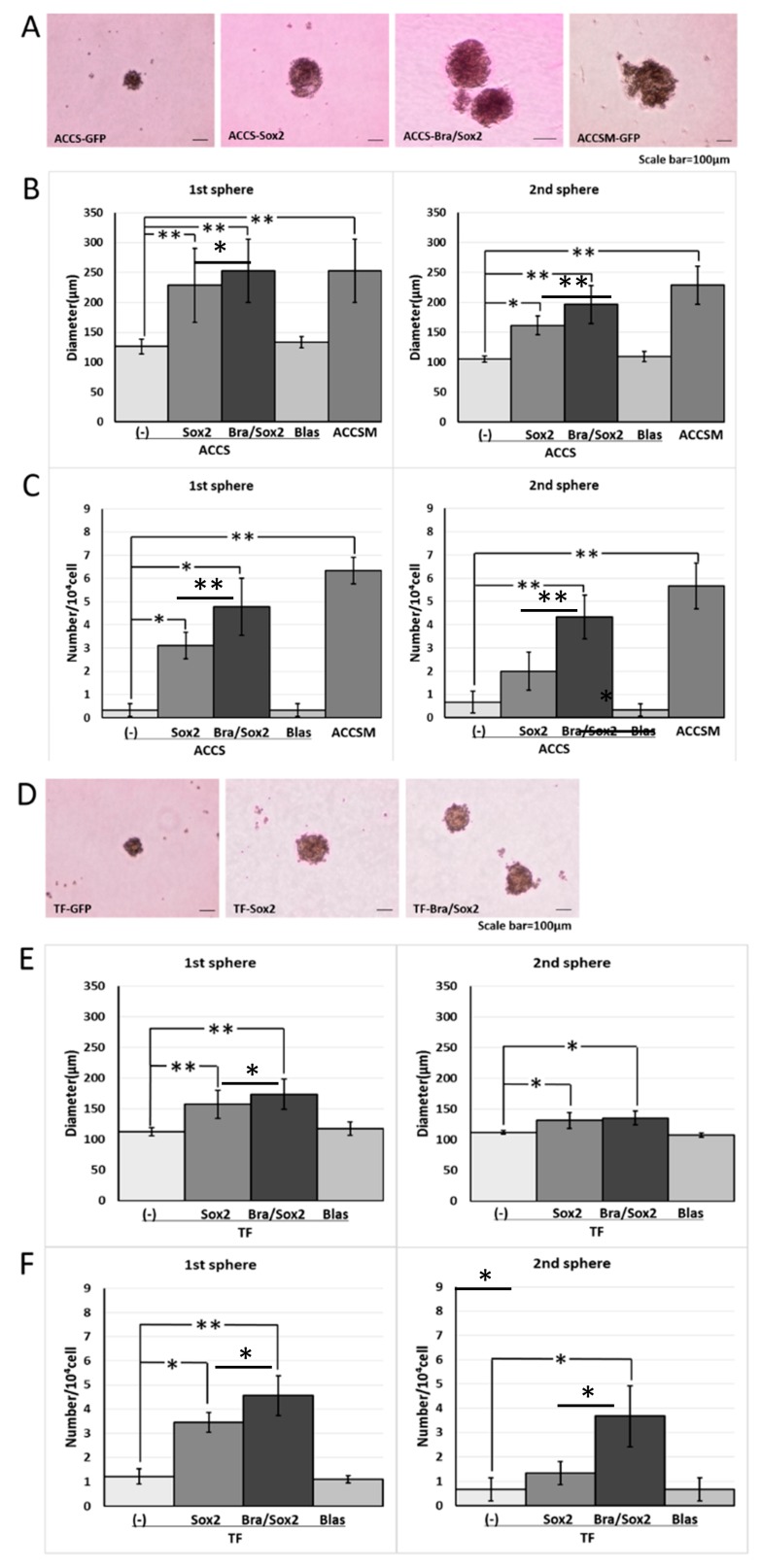
Effects of *BRACHYURY* and *SOX2* transfection on the sphere-forming ability of ACCS (adenoid cystic carcinoma) and TF (squamous cell carcinoma) cells. ACCS-Sox2, ACCS-Blasticidin (Blas), ACCS-Bra/Sox2, TF-Sox2, TF-Blasticidin (Blas), and TF-Bra/Sox2 cells were cultured, and the sphere-forming ability was quantified as described in the legend of Figure 1. Images of phase contrast microscope (**A**,**D**). Sphere diameters (*P* < 0.0001, ANOVA) (**B**,**E**), and Sphere numbers (*P* < 0.0001, ANOVA) (**C**,**F**) were analysed as described in the legend of Figure 1. The experiments were performed in triplicate, and the data were calculated as mean ± SD. Statistical significance of differences was analysed using the Student’s *t* test. * *P* < 0.05, ** *P* < 0.01.

We previously reported that *SOX2* knockdown partially inhibits CSC and EMT phenotypes of ACCS-M GFP cells [11]. The data from forced expression and a knockdown of *BRACHYURY* in oral carcinoma cell lines suggests that Brachyury may need a partner to drive the expression of the CSC phenotype. Therefore, we next analysed the effect of forced co-expression of *BRACHYURY* and *SOX2* on the self-renewal capacity. To this end, we established *SOX2* transfectants and *BRACHYURY* + *SOX2* co-expression transfectants of ACCS-GFP and TF-GFP cells: ACCS-Sox2, ACCS-Bra/Sox2, TF-Sox2, and TF-Bra/Sox2. Forced expression of *SOX2* enhanced sphere formation (the number of spheres) in the primary sphere assay compared to parental cells ACCS-GFP (9.3-fold) and TF-GFP (2.8-fold), but failed to increase the number of spheres in the secondary sphere assay. In contrast, co-expression of *BRACHYURY* and *SOX2* enhanced sphere formation in both the primary and the secondary sphere assays, with respect to both the diameter and the number of spheres, in ACCS and TF cells. This enhancement reached the level of ACCS-M GFP cells (Figure 5). Forced expression of *SOX2* or co-expression of *BRACHYURY* and *SOX2* did not change the cell proliferation rate on cell culture dish (data not shown).

### 2.4. Forced Co-Expression of BRACHYURY and SOX2 Induced EMT-Related Markers and Stem Cell Markers in Oral Cancer Cell Lines

**Figure 6:**
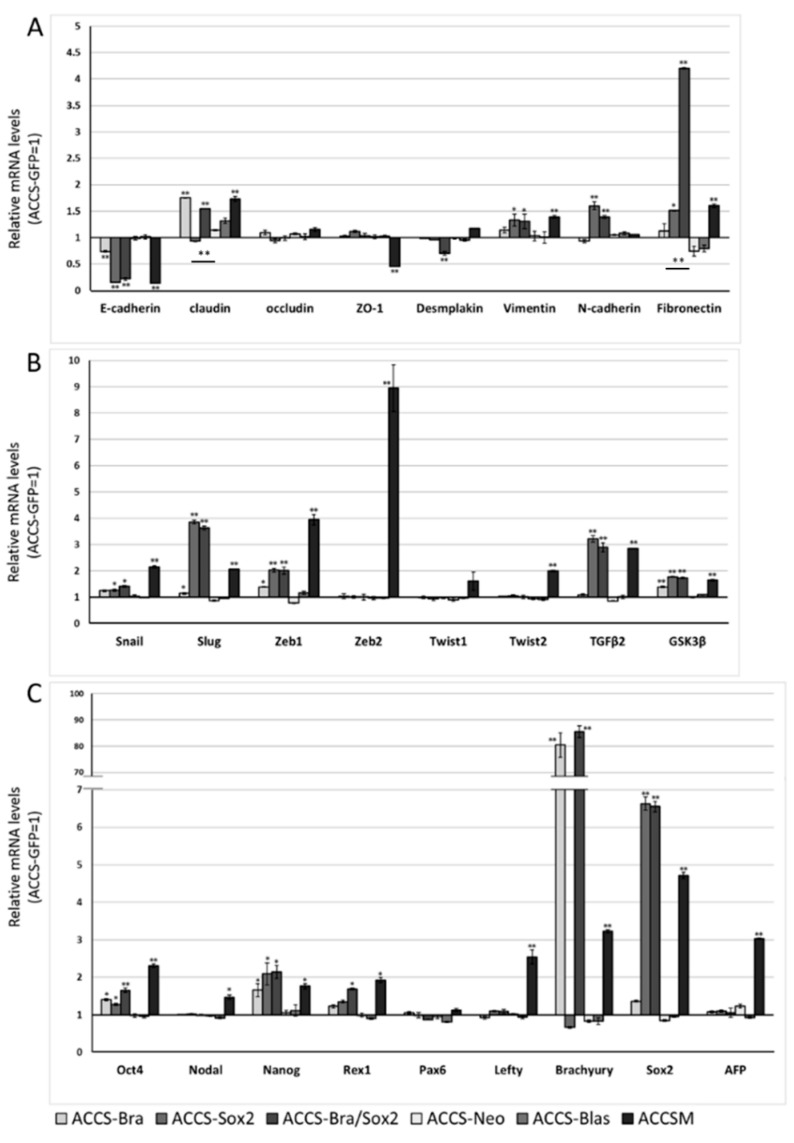

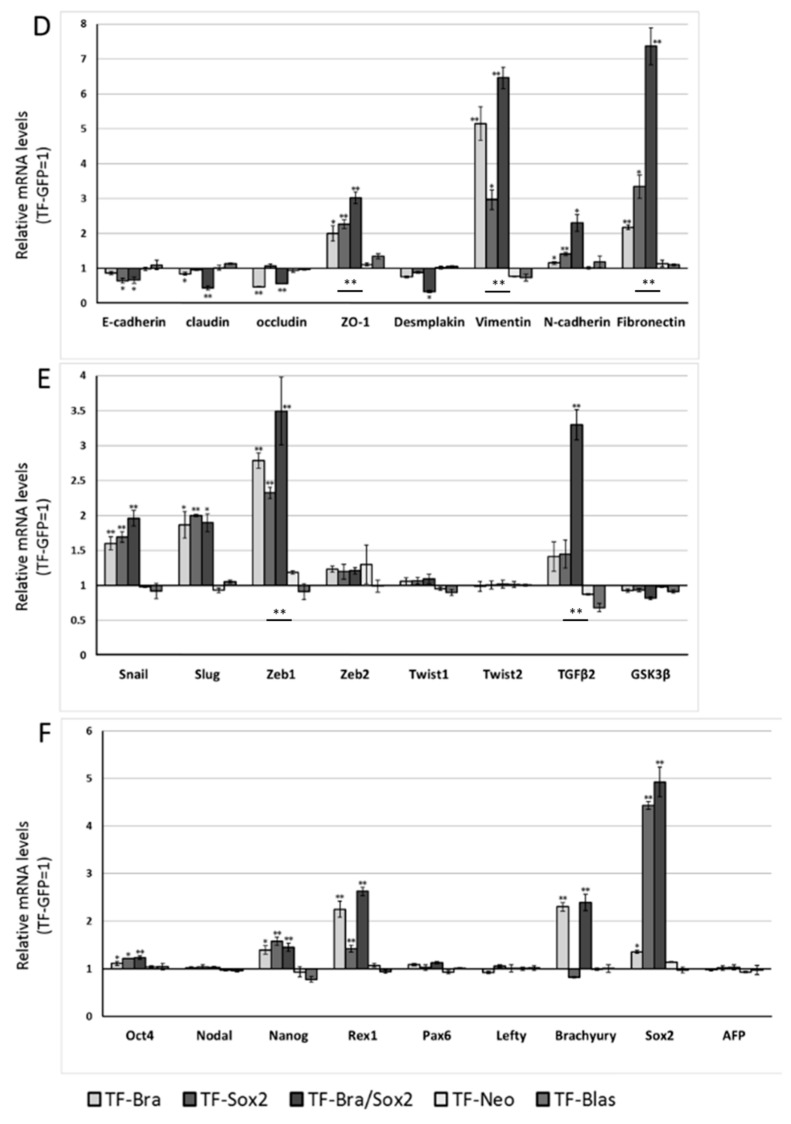
Effects of *BRACHYURY* and *SOX2* transfection on gene expression related to epithelial–mesenchymal transition (EMT) and cancer stem cell (CSC) phenotype in ACCS and TF cells. The mRNA expression levels of the indicated genes in ACCS (adenoid cystic carcinoma) and TF (squamous cell carcinoma) cells and in derivative clones were quantified using real-time RT-PCR. Each mRNA level was compared with that in ACCS-GFP or TF-GFP cells, and the data are shown in arbitrary units as relative mRNA levels (ACCS-GFP, TF-GFP = 1.0). The expression levels of EMT-related genes (**A**,**B**,**D**,**E**), stem cell markers, and differentiation markers (**C**,**F**) are shown. The experiments were performed in triplicate, and the data were calculated as mean ± SD. Statistical significance of differences was analysed using the Student’s *t* test. * *P* < 0.05, ** *P* < 0.01.

We next analysed the effect of *SOX2* and co-expression of *BRACHYURY* and *SOX2* on EMT-related markers and stem cell markers in ACCS (Figure 6A–C) and TF (Figure 6D–F) cell lines in the same way as shown in Figure 3. The expression levels of EMT-related genes (Figure 6A,D), stem cell markers (Figure 6B,E), and differentiation markers (Figure 6C,F) are shown. In ACCS cells, forced expression of SOX2 caused a significant increase in mRNA expression of *VIMENTIN, N-CADHERIN, FIBRONECTIN, SNAIL, SLUG, ZEB1, TGF-β2, GSK3β, OCT4, and NANOG* and caused a decrease in *E-CADHERIN* mRNA (Figure 6A–C). In TF cells, forced expression of SOX2 caused a significant increase in mRNA expression of *VIMENTIN*, *N-CADHERIN*, *FIBRONECTIN*, S*NAIL*, *SLUG*, *ZEB1*, *OCT4*, *REX1*, and *NANOG* and decreased *E-CADHERIN* expression (Figure 6D–F). The pattern of changes was nearly the same in the 2 cell lines. Co-expression of *BRACHYURY* and *SOX2* had synergistic effects on the enhancement of mRNA expression of *FIBRONECTIN*, *SNAIL*, *SLUG*, *ZEB1*, and *TGF-β2* in both cell lines. These synergistic effects on *FIBRONECTIN* and *TGF-β2* were especially strong.

### 2.5. Artificial CSC-Like Cells Express an Invasive Phenotype In Vitro

**Figure 7:**
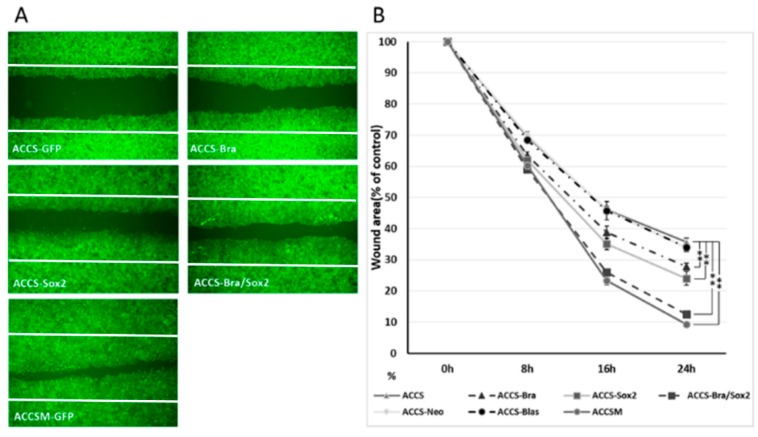

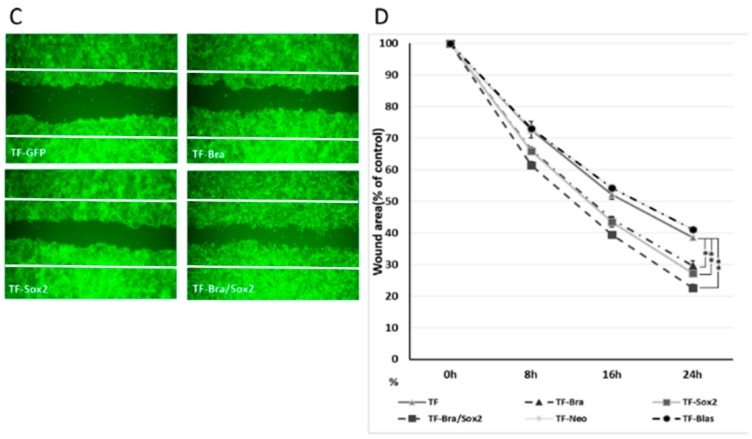
Effects of *BRACHYURY* and *SOX2* transfection on cell migration of ACCS and TF cells. (**A**,**C**) ACCS (adenoid cystic carcinoma) and TF (squamous cell carcinoma) cells and derivative clones were seeded at 3 × 10^5^ cells per well in a 6-well plate and incubated for 24 h so that a confluent monolayer of cells could form. After 24 h, scratch wounds were inflicted on the confluent monolayer of cells (white line) and incubation was continued for a further 24 h. Randomly chosen wound fields were photographed under a fluorescence microscope in the course of 24 h every 8 h. (**B**,**D**) The wound areas were evaluated using the following formula: wound area (% of control) = (wound area after the indicated period/initial wound area) × 100. The experiments were performed in triplicate, and the data were calculated as mean ± SD and ANOVA (*P* < 0.0001). Statistical significance of differences was analysed using the Student’s *t* test. * *P* < 0.05, ** *P* < 0.01.

The above results suggested that co-expression of *BRACHYURY* and *SOX2* induces the CSC phenotype. We then tested whether these artificial CSC-like cells show signs of invasiveness in vitro. The effect of forced expression of *BRACHYURY* or/and *SOX2* on cell migration of oral cancer cells in vitro was analysed using a wound healing assay (Figure 7). Cell migration of ACCS-M GFP cells was approximately 2 times faster than that of ACCS GFP cells. Expression of *BRACHYURY* or *SOX2* increase the cell migration (*P* < 0.01) but failed to reach the level of ACCS-M. 

Co-expression of *BRACHYURY* and *SOX2* increased cell migration almost to the level of the CSC-like cells. There was no significance between ACCS-M GFP and ACCS Bra/Sox 2 (*P* = 0.183) (Figure 7A,B). Cell migration of TF-Bra/Sox2 cells was approximately 3 times faster than that of TF GFP cells (*P* = 0.001; Figure 7C,D).

**Figure 8:**
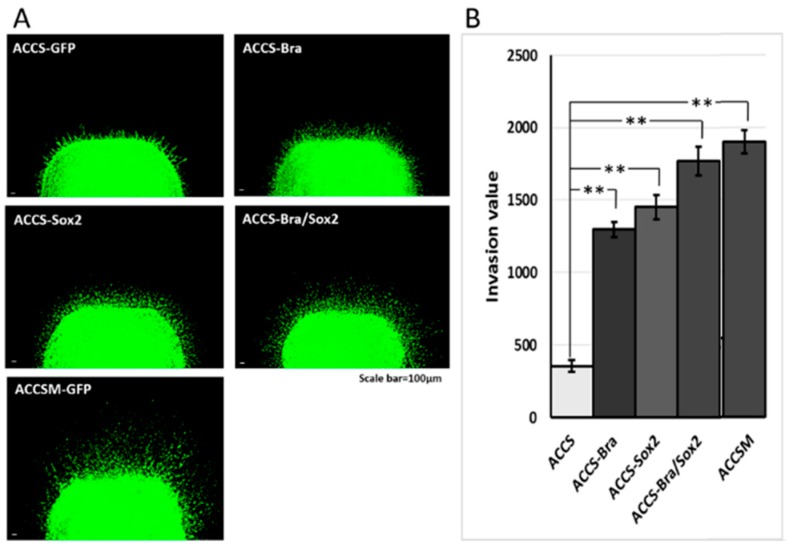

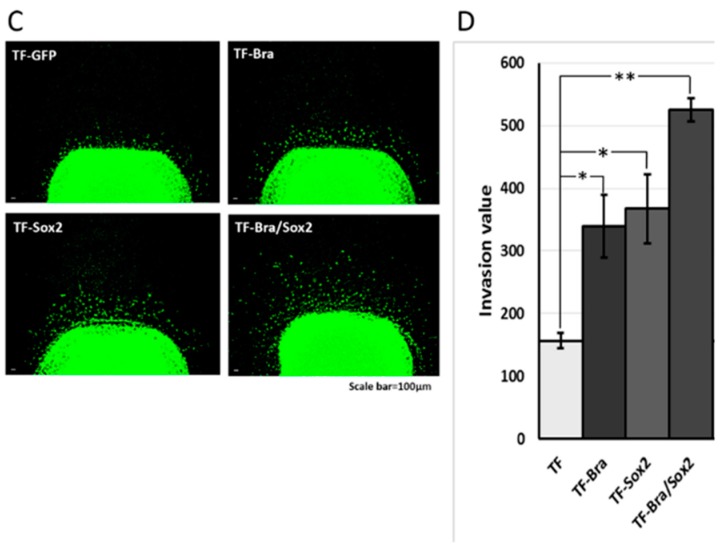
Effects of *BRACHYURY* and *SOX2* transfection on cell invasiveness of ACCS and TF cells. One million ACCS and TF cells each were pelleted and solidified as described in Materials and Methods. The collagen-embedded tumour cells were embedded in the collagen-embedded fibroblasts and allowed to invade into artificial stroma. After 7 days, tumour dissemination was observed under a fluorescence microscope (**A**,**C**). Tumour dissemination from the tumour cell pellet was evaluated in 5 randomly selected standardised rectangular light fields (500 × 100 μm), and the values were summed as described in Materials and Methods (**B**,**D**). The experiments were performed in triplicate, and the data were calculated as mean ± SD and ANOVA (*P* < 0.0001). Statistical significance of differences was analysed using the Student’s *t* test. * *P* < 0.05, ** *P* < 0.01.

We next analysed the effect of forced expression of *BRACHYURY* or/and *SOX2* on cell invasiveness in vitro using a tumour cell dissemination assay that we established previously [23]. In this assay, carcinoma cells invade to artificial stroma (fibroblast embedded collagen gel) are visualised as fluorescent spots migrating from artificial primary cancer nest (cancer cell pellet solidified with collagen gel). Cancer cell invasiveness is evaluated by the number of invasive cells and their distance from the artificial primary cancer nest. As shown in Figure 8A, ACCS-M GFP cells invaded into artificial stroma very aggressive manner. Co-expression of *BRACHYURY* and *SOX2* strongly enhanced the invasiveness of ACCS-GFP cells to the level of the aggressive ACCS-M GFP cells *(P* = 0.223*)* (Figure 8A,B). Figure 8B shows the comparison of invasiveness among the ACCS cell lines. Relative invasiveness values (ACCS GFP = 1.0 arbitrary unit) were 3.7 (ACCS-Bra), 4.1 (ACCS-Sox2), 5.0 (ACCS-Bra/Sox2), and 5.6 (ACCS-M GFP). Expression of *BRACHYURY* or *SOX2* increase the cell invasiveness (*P* < 0.01) but failed to reach the level of ACCS-M.

Invasiveness of TF-GFP (squamous cell carcinoma) cells was strongly enhanced by co-expression of *BRACHYURY* and *SOX2* (Figure 8C). Figure 8D shows comparison of the invasiveness among the squamous cell carcinoma cell lines. Relative invasiveness values (TF GFP = 1.0) were 2.2 (TF-Bra), 2.3 (TF-Sox2), and 3.3 (TF-Bra/Sox2).

## 3. Discussion

Metastasis is a most life-threatening event which directly influencing prognosis in cancer progression. Accumulated evidences suggest that cancer invasion and metastasis is strongly regulated by EMT in cancer cells [24,25,26]. Similarly, CSCs have attracted more and more attention as targets for cancer therapy because they have been reported to exhibit chemo- and radio-resistance [16,17,27,28,29]. More recently, the direct link between EMT and CSC phenotype was reported [6,24,30,31], but the regulatory mechanisms behind cancer stemness and EMT are still unclear.

We recently showed that CSC phenotypes, including EMT, could be blocked by knocking down the transcriptional factor *BRACHYURY* in vitro and in vivo [11]. We also showed downregulation of EMT-related genes, stem cell markers, and loss of tumourigenic and metastatic potential in vivo as a result of knockdown of *BRACHYURY* in a CSC-like cell line, ACCS-M GFP [11]. This finding suggests that *BRACHYURY* is a key regulator of the CSC phenotype. Sarkar et al. [32] also reported that a *BRACHYURY* knockdown via siRNA resulted in downregulation of expression of *CD44*, *CD166*, *CD133*, *ALDH1*, and *NANOG* in colon cancer, and they concluded that *BRACHYURY* participates in establishment of CSC characteristics by inducing *NANOG* expression. In the present study, we showed that overexpression of *BRACHYURY* upregulated *NANOG* expression slightly, but failed to promote self-renewal capacity not only in ACCS GFP (adenoid cystic carcinoma) cells but also in TF GFP, a squamous cell carcinoma cell line. Fernando et al. [21] reported that forced expression of *BRACHYURY* caused induction of EMT (gain of mesenchymal markers and loss of epithelial markers) and increased in cell migration and invasion in human carcinoma cell line. In this regard, our results completely match their findings. It is thought that *NANOG* is not absolutely required for self-renewal of embryonic stem cells but is required for pluripotency [33]. In line with this notion, in our present experiments, *BRACHYURY* overexpression promoted only the EMT phenotype, a type of invasive phenotype. Some authors proposed to classify EMT into 3 subtypes based on biological status with biomarkers [34,35]. Type 1; EMT associated with organ development, Type 2; EMT related to wound healing and regeneration. EMT in cancer progression and metastasis is categorized as type 3 EMT and specific signaling or biomarkers has yet to be determined. *TGF-β* would be the most responsible factor for induction of EMT by inducing EMT-inducing transcription factors in cancer cells, notably *SNAIL*, *SLUG*, *ZEB1*, *TWIST*, *GOOSECOID*, and *FOXC2* [36].

*SOX2* (SRY Sex Determining Region Y-Box2) is a member of the Sox (SRY-related HMG box) family of transcription factors. *SOX2* maintains stemness and pluripotency of normal stem cells by regulating gene transcription. Recent reports revealed the association between *SOX2* and EMT [37,38]. In line with these reports, *SOX2* overexpression resulted in specific changes in EMT of ACCS-Sox2 cells in the present work. It is also well known that *SOX2* promotes metastasis of breast and prostate cancer cells via activation of the Wnt/β-catenin pathway [39]. Sox2 protein binds to the promoter region of β-catenin and regulates its expression. In addition, Sox2 protein regulates the protein expression of downstream elements in Wnt signaling, DKK3, DVL1, and DVL3 [39]. Aberrant activation of Wnt/β-catenin signaling has been described in a variety of cancers, and inhibition of Wnt/β-catenin by DKK-1 resulted in diminution of the self-renewal capacity of a gastric cancer cell line [40]. Overexpression of *SOX2* increased the self-renewal capacity in both ACCS GFP and TF GFP cells in the present work. Nonetheless, the self-renewal capacity of ACCS-Sox2 cells was approximately 50% in size and 30% in number compared to ACCS-M GFP cells, even though *SOX2* expression was 150% higher in ACCS-Sox2 cells. These data suggest that *SOX2* promotes EMT and self-renewal capacity, but it is not a key player in these processes.

Several factors have been reported as indispensable for stem cell maintenance and differentiation. For example, *OCT3/4*, *SOX2*, *KLF-4*, and *C-MYC* are necessary for expression of the phenotype of induced pluripotent stem cells in human and mouse fibroblasts [41,42]. These factors have been reported to physically or synergistically interact with each other during development [43]. The same kind of protein or gene network may exist in cancer stem cells. Chen et al. reported that silencing of the *SOX2* gene reduces the tumourigenic potential of a lung cancer cell line (A549 cells) with downregulation of *C-MYC*, *WNT1*, *WNT2*, and *NOTCH1* in mice; these authors uncovered 246 additional target cancer genes of *SOX2* [44]. In the present work, we found significant upregulation of *TGF-β* and fibronectin as a result of simultaneous overexpression of *SOX2* and *BRACHYURY*. This synergistic effect of *SOX2* and *BRACHYURY* in the promotion of the invasive phenotype of CSCs, especially when it comes to *TGF-β* and *FIBRONECTIN* expression, could be explained by the same kind of protein or signaling network as mentioned above. 

*SOX2* activates the Wnt/β-catenin pathway and causes β-catenin activation [39], and the target gene of this activated β-catenin is *BRACHYURY* [45]. It was shown that *BRACHYURY* promotes the EMT phenotype and positively relate each other with *TGF-β* in mesenchymal-like tumour cells [46,47]. *BRACHYURY* stimulates *TGF-β1* expression, and overexpressed *TGF-β1* induces *SOX2* in turn [48]. *SOX2* also positively regulates *FIBRONECTIN* expression [49], and *FIBRONECTIN* enhances the EMT phenotype and self-renewal capacity [50]. Therefore, a potentially unlimited autocrine loop is expected to be established among *SOX2*, *BRACHYURY*, *TGF-β*, and *FIBRONECTIN*. As discussed above, *BRACHYURY* mainly plays a role in EMT, and *SOX2* is involved in EMT and self-renewal. Their synergistic effect, however, may be more crucial because the interaction of the *SOX2* and *BRACHYURY* gene networks activates a vast network of genes/proteins that drives the development of cancer invasiveness. In other words, the closer to the centre of the network (*SOX2* and *BRACHYURY*) a target factor is, the greater the impact of its inactivation on EMT and cancer stemness. This is why silencing of *BRACHYURY* is observed to be so effective.

In this study, we showed that co-expression of *BRACHYURY* and *SOX2* promotes both EMT and self-renewal phenotypes in ACCS-GFP cells; phenotypes that resemble ACCS-M GFP, the CSC-like cell line from in vivo selection. Nonetheless, the patterns of the expression of some genes (e.g., *ZEB2*) are still different between ACCS-M GFP cells and ACCS Bra/Sox2. We could not enhance *ZEB2* expression by co-expressing *BRACHYURY* and *SOX2*. The role of *ZEB2* in CSCs is not clear, and further research is needed to identify which factor(s) control *ZEB2* expression and its function in cancer stemness. 

Our results also revealed that the regulation of CSC related genes by *BRACHYURY* and *SOX2* is different by cancer cell origin (stratified squamous epithelial cells and salivary gland cell). Effect of *BRACHYURY* and *SOX2* expression on EMT and pluripotency related genes varied in degree of changes between cell types. As mentioned above, *BRACHYURY* and *SOX2* were not sufficient for initiation of cancer stem cell property, especially pluripotency related genes. However, it is also true that *BRACHYURY* is required for tumourigenesis and metastasis, the most important property for cancer stem cell in vivo. Because it has been described in our previous study, tumourigenesis and metastasis of ACCSM cell were prevented completely by silencing of *BRACHYURY* and partially by silencing of *SOX2* in vivo [11]. It was also confirmed that protein expression of Brachyury strongly correlates with EMT and metastasis involvement in oral cancer patients [5].

These results will provide important clues as to how the CSC phenotype is developed via accumulating alterations in gene networks and why this phenomenon is very similar to developmental processes with respect to their effects, but is completely different with respect to their mechanisms. When all pieces of the puzzle are in place, researchers will be able to explain why cells expressing the EMT phenotype sometimes fail to metastasise [51,52,53] and why cancer stemness exhibits heterogeneity. 

We conclude that *BRACHYURY* and *SOX2* synergistically promote EMT (invasiveness) and self-renewal property, and one of the members of cancer stemness related genes. This new knowledge is expected to lead to more specific and effective therapeutic targets in oral cancer, which may include *SOX2* and *BRACHYURY*, either alone or in combination.

## 4. Materials and Methods 

### 4.1. Cells and Culture

ACCS, ACCS GFP, and ACCS-M GFP (human oral adenoid cystic carcinoma cell lines) were established and reported previously in our laboratory [22]. In brief, ACCS-GFP was established from the parental cell line ACCS by transfecting green fluorescence protein (GFP) gene. These cell lines had similar morphological characteristics, growth rates, and tumourigenicity in vitro and in vivo. Tumourigenicity of ACCS-GFP was similar to the parental ACCS, (22.2% incidence). ACCS-GFP cells were injected to the tongues of nude mice and the developed tumour was clearly detectable with green fluorescence under excitation light. ACCS-M GFP cells was established by in vivo selection process repeatedly. ACCS-M GFP cells exhibited high tumourigenicity (100% incidence) and spontaneous metastases to submandibular lymph nodes (100% incidence). 

SCCTF is a *BRACHYURY*- and *SOX2*-negative human oral squamous cell carcinoma cell line. TF-GFP and TF-Neo transfectant cell lines were generated via transfection with the pEGFP-N1 vector and a control vector (Clontech; Palo Alto, CA, USA) for visualisation, using Lipofectamine LTX (Invitrogen; Carlsbad, CA, USA) according to the manufacturer’s instructions. Colony selection after individual transfection procedure was performed using resistance to neomycin (G418; Sigma-Aldrich; St. Louis, MO, USA). The cell lines were maintained in Dulbecco’s modified Eagle medium (DMEM; Sigma-Aldrich, St. Lois, MO, USA) supplemented with 10% fetal bovine serum (FBS; ICN Biomedicals; Aurora, OH, USA), 2 mM L-glutamine, penicillin G, and streptomycin in a humidified incubator with an atmosphere of 5% CO_2_ at 37 °C.

### 4.2. Transfection and Knockdown of BRACHYURY and SOX2

For forced expression of *BRACHYURY*, full-length human Brachyury cDNA was cloned into the pCMV6 vector (ORIGENE Technologies Inc., Rockville, MD, USA), resulting in the pCMV6-Brachyury vector. ACCS-Bra and TF-Bra cells were generated via transfection of the pCMV6-Brachyury vector into ACCS-GFP and TF-GFP cells, respectively. As controls, ACCS-Neo and TF-Neo cells were generated by means of transfection of the pCMV6-Entry vector into ACCS-GFP and TF-GFP cells, respectively.

For forced expression of *SOX2*, full-length human *SOX2* was cloned into the pUNO1 vector (Invitrogen, Carlsbad, CA, USA), resulting in the pUNO1-hSox2 vector. ACCS-Sox2 and TF-Sox2 cells were generated via transfection of the pUNO1-hSox2 vector into ACCS-GFP and TF-GFP cells, respectively. As controls, ACCS-Blas and TF-Blas cells were generated by means of transfection of the pUNO-mcs vector into ACCS-GFP and TF-GFP cells, respectively.

ACCS-Bra/Sox2 and TF-Bra/Sox2 cells were generated via co-transfection of the pCMV6-Brachyury and pUNO1-hSox2 vectors into ACCS-GFP and TF-GFP cells, respectively. The transfection was performed using Lipofectamine LTX (Invitrogen) according to the manufacturer’s instructions.

Gene knockdown experiment was performed on Brachyury express ACCSM-GFP cells by transfecting an shRNA lentiviral plasmid (pLKO.1-puro; Sigma-Aldrich) using Lipofectamine LTX (Invitrogen). Control cells (ACCS-sh.control and ACCS-M-sh.control) were generated by transfection of ACCS GFP and ACCS-M GFP cells with the pLKO.1-puro Control Vector (Sigma-Aldrich). Brachyury knockdown cells (ACCS-shBra and ACCS-M-shBra) were generated by transfection of ACCS GFP and ACCS-M GFP cells with pLKO.1-puro/sh.Brachyury (Sigma-Aldrich). The expression level of Brachyury in shRNA-transfected ACCS cells was monitored using real-time RT-PCR. All transfected cells were maintained in DMEM containing 10% FBS and 2 μg/mL puromycin (Sigma-Aldrich).

### 4.3. Real-Time RT-PCR

Real-time RT-PCR was employed to quantify the mRNA expression levels of the indicated EMT-related genes, embryonic stem cell markers, and differentiation markers in ACC cells and TF cells. Total RNA was extracted and purified using TRIzol Reagent (Invitrogen). The first-strand cDNA synthesis and amplification of target mRNA for quantification were performed using a real-time PCR system: LightCycler FastStart DNA Master SYBER Green 1 kit (Roche Diagnostics; Mannheim, Germany). The mRNA levels were quantified in triplicate. The PCR cycling conditions are as follows; 10 min at 95 °C for 1 cycle followed by 45 cycles at 95 °C for 30 s, 60 °C for 30 s, and 72 °C for 60 s. Dissociation curve analysis, normalisation by β-actin mRNA levels and quantification of target mRNA expression level were analysed using the LightCycler 2.0 System software package (Roche Applied Science; Indianapolis, IN, USA). The specific primers for EMT, stem cell markers, and differentiation markers are shown in Table 1. All primers were purchased from NIHON Gene Research Laboratories, Inc. (Sendai, Japan).

### 4.4. The Sphere-Formation Assay

Cultured cells were recovered and plated for floating cultures at a density of 5 × 10^4^ cells/mL in 60-mm non-coated dishes with serum-free DMEM containing basic fibroblast growth factor (bFGF, 40 ng/mL) and epidermal growth factor (EGF, 20 ng/mL). The cells were incubated in a humidified atmosphere at 37 °C and 5% CO_2_, and bFGF and EGF were added to the medium every other day during culture period. After 10 days, the diameters of each cell sphere were measured, and the numbers of sphere with a diameter >100 μm were counted as primary spheres. For passaging of the primary spheres, the spheres were collected by centrifuge and treated with 0.05% trypsin/0.02% ethylendiaminetetraacetic acid (EDTA) and dissociated into single cells. The dissociated cells were plated to 24-well culture plates at a density of 10^4^ cells/mL in a serum-free medium and allowed to grow for further 10 days in a serum-free medium to obtain secondary spheres.

### 4.5. The Wound Healing Assay

Cancer cells were plated at the density of 6 × 10⁵ cells per well in a 6-well plate (BD Falcon) in DMEM with 10% FBS and allowed to attach for 24 h to obtain confluent monolayer of cells. Then monolayer cells were treated with 25 µg/mL of Mitomycin C for 25min at 37 °C to prevent cell growth. The confluent monolayer of cells is scratched to make wounds using a plastic pipette tip (200 µL), and the cells were washed with phosphate-buffered saline (PBS) and arrowed to migrate for 24 h. Randomly chosen wound fields were photographed under a fluorescence microscope (BZ-8000; Keyence; Osaka, Japan) every 8 h for 24 h. The wound areas were analysed using the following formula:

Wound area (% of control) = (wound area after the indicated period/initial wound area) × 100(1)

### 4.6. Evaluation of Tumour Dissemination from the Primary Cancer Nest

Evaluation of tumour dissemination from a primary cancer nest was performed as described previously [23]. One million cells were pelleted and resuspended in 25 µL of DMEM and 25 µL of type I collagen (AteloCell; IAC-30; Koken; Tokyo, Japan) to form a solid cell cluster. The collagen-embedded tumour cell pellets were incubated to solidify for 30 min at 37 °C in a 1.5-mL microcentrifuge tube. Five hundred microliters of type I collagen containing fibroblasts (10^5^ cells/mL) were added to a 6-well plate (BD Falcon, Franklin Lakes, NJ, USA). The collagen-embedded tumour cells were embedded in the collagen-embedded fibroblasts and incubated to solidify for 30 min at 37 °C. DMEM was added on top of the collagen gels, and the incubation was continued. After 7 days of culture, tumour migration was observed as green fluorescence under a fluorescence microscope (BZ-8000; Keyence, Osaka, Japan). Cancer cell invasiveness was evaluated by tumour dissemination from the tumour cell pellet (mimics a primary tumour nest). Evaluation were made by measuring the distance of all cells from the edge of the nest in 5 randomly selected standardised rectangular light fields (500 µm × 100 µm), and the values were summed (invasion value).

### 4.7. Statistical Analysis

All data were calculated as mean ± SD and processed using analysis of variance (ANOVA) for multiple group comparison. When ANOVA is significant, two groups comparison using the Student’s *t*-test is performed. Statistical analyses were calculated by means of the statistical software SPSS 13.0. Statistical significance was assumed at *P <* 0.05*.*

## Figures and Tables

**Table 1:** Primers used in real-time RT-PCR.

Gene (Human)	Primer Sequence
*E-CADHERIN*	(F) 5′ CAA CTG GAC CAT TCA GTA CAA C 3′ (R) 5′ TCC ATG AGC TTG AGA TTG AT 3′
*CLAUDIN*	(F) 5′ GAC AAC ATT CAC TGCC TCA GG 3′ (R) 5′ TTC ACA TTT GGT GAT TCT CG 3′
*OCCULUDIN*	(F) 5′ CTC GAA GAA AGA TGG ACA GGT 3′ (R) 5′ GCC ATG GGA CTG TCA ACT C 3′
*ZO-1*	(F) 5′ CGA AGG AGT TGA GCA GGA AAT CT 3′ (R) 5′ TCC ACA GGC TTC AGG AAC TTG 3′
*DESMOPLAKIN*	(F) 5′ ACC GCT GGC AAA GGA TAG AT 3′ (R) 5′ CCA CTT GCA GAA AGC CTG AT 3′
*VIMENTIN*	(F) 5′ ATT CAC TCC CTC TGG TTG ATA C 3′ (R) 5′ CGT GAT GCT GAG AAG TTT CG 3′
*N-CADHERIN*	(F) 5′ GAC AAC ATT CAC TGC TCA GG 3′ (R) 5′ TTC ACA TTT GGT GAT TCT CG 3′
*FIBRONECTIN 1*	(F) 5′ CAA TGC CAG GAT TCA GAG AC 3′ (R) 5′ CTT CGA CAG GAC CAC TTG AG 3′
*SNAIL*	(F) 5′ TCC ACA AGC ACCAAG AGT C 3′ (R) 5′ ATG GCA GTG AGA AGG ATG TG 3′
*SLUG*	(F) 5′ ACT GCT CCA AAA CCT TCT CC 3′ (R) 5′ TGG TCA GCA CAG GAG AAA ATG 3′
*TWIST 1*	(F) 5′ CTC AGC TAC GCC TTC TCG 3′ (R) 5′ ACT GTC CAT TTT CTC ATT CTC TG 3′
*TWIST 2*	(F) 5′ AGG AGC TCG AGA GGC AG 3′ (R) 5′ CGT TGA GCG ACT GGC TG 3′
*ZEB1*	(F) 5′ CTC ACA CTC TGG GTC TTA TTC TC 3′ (R) 5′ GTC TTC ATC CTC TTC CCT TGT C 3′
*ZEB2*	(F) 5′ AAA GGA GAA AGT ACC AGC GG 3′ (R) 5′ AGG AGT CGG AGT CTG TCA TAT C 3′
*TGFB2*	(F) 5′ TTA ACA TCT CCA ACC CAG CG 3′ (R) 5′ TCC TGT CTTTAT GGT GAAGCC 3′
*GSK3B*	(F) 5′ GGT CTA TCT TAA TCT GGT GCT GG 3′ (R) 5′ AGG TTC TGC GGT TTA ATA TCC C 3′
*NODAL*	(F) 5′ ACC CAG CTG TGT GTA CTC AA 3′ (R) 5′ TGG TAA CGT TTC AGC AGA C 3′
*OCT4*	(F) 5′ TAT CGA GAA CCG AGT GAG AG 3′ (R) 5′ TCG TTG TGC ATA GTC GCT 3′
*PAX6*	(F) 5′ GGC GGA GTT ATG TAT ACC TAC 3′ (R) 5′ CTT GGC CAG TAT TGA GAC AT 3′
*REX1*	(F) 5′ AAA CGG GCA AAG ACA AGA 3′ (R) 5′ GCT CAT AGC ACA CAT AGC CAT 3′
*LEFTY*	(F) 5′ TGT ATC CAT TGA GCC CTC T 3′ (R) 5′ CAG GAA ATG GAA GGA CAC A 3′
*NANOG*	(F) 5′ ACC CAG CTG TGT GTA CTC AA 3′ (R) 5′ GCG TCA CCA TTG CTA TT 3′
*BRACHYURY*	(F) 5′ TGC TGC AAT CCC ATG ACA 3′ (R) 5′ CGT TGC TCA CAG ACC ACA 3′
*SOX2*	(F) 5′ TGG GTT CGG TGG TCA AGT 3′ (R) 5′ CTC TGG TAG TGCTGG GAC A3′
*AFP*	(F) 5′ CTG CAA ACT GAC CAC GCT 3′ (R) 5′ TGA GAC AGC AAG CTG AGG AT 3′

## Abbreviations

EMT	Epithelial–mesenchymal transition
CSCs	Cancer stem cells
shRNA	Small hairpin RNA
GFP	Green fluorescence protein
EDTA	Ethylendiaminetetraacetic acid
